# Laparoscopic sleeve gastrectomy with adrenalectomy, feasibility, safety and outcome

**DOI:** 10.1093/jscr/rjac130

**Published:** 2022-05-05

**Authors:** Awadh Alqahtani, Mohammad Almayouf, Srikar Billa, Hadeel Helmi

**Affiliations:** Department of Surgery, College of Medicine, King Saud University, Riyadh, Saudi Arabia; Department of Surgery, College of Medicine, Prince Sattam bin Abdulaziz University, Al-Kharj, Saudi Arabia; Department of Surgery, Dr. Sulaiman Al-Habib Hospitals, Riyadh, Saudi Arabia; Department of Surgery, College of Medicine, King Saud University, Riyadh, Saudi Arabia

## Abstract

Concomitant surgery is an attractive option because of convenience. To our knowledge, this is the first study reporting concomitant laparoscopic sleeve gastrectomy (LSG) and laparoscopic right adrenalectomy. A retrospective review of three patients with obesity and a unilateral adrenal mass was conducted. The demographics, workup, surgical technique and outcome were presented. Patient 1 had a body mass index (BMI) of 41 kg/m^2^, diabetes mellitus (DM), hypertension (HTN) and a right adrenal pheochromocytoma. Patient 2 had a BMI of 40 kg/m^2^, insulin-dependent DM, uncontrolled HTN, chronic kidney disease, ischemic heart disease and an aldosterone secreting right adrenal adenoma. Patient 3 had a BMI of 41 kg/m^2^, dyslipidemia, HTN and gout. All patients underwent concomitant LSG and laparoscopic adrenalectomy (LA). LSG and LA is a feasible and safe concomitant surgery when performed under specific measures with minimal morbidity and more convenience.

## Teaser

• Laparoscopic sleeve gastrectomy and laparoscopic adrenalectomy are not commonly performed concomitantly.

• Under certain conditions, this set of procedures can be done concomitantly under certain conditions.

• Concomitant surgery is effective and convenient with minimal morbidity.

## INTRODUCTION

Metabolic surgery is effective in managing obesity and its accompanying health comorbidities [1–3]. Thus, the rate of metabolic surgery has risen dramatically in the last years, which increases the chance of concomitant surgery [4]. The most common concomitant procedure with metabolic surgery is laparoscopic cholecystectomy. This set of surgeries was extensively investigated from a safety and outcome perspective, which showed minimal morbidity [5, 6]. Conversely, the literature is limited regarding metabolic surgery and concomitant laparoscopic adrenalectomy (LA). Laparoscopic left adrenalectomy (LLA) has been previously described to be done simultaneously with laparoscopic sleeve gastrectomy (LSG) or gastric bypass surgery. However, to the best of our knowledge, we are reporting the first cases to combine LSG with laparoscopic right adrenalectomy (LRA). This study describes the management of patients with obesity and a unilateral adrenal mass, surgical techniques and the outcome following concomitant surgery.

## CASE SERIES

This is a retrospective review of three patients who were having obesity and a unilateral adrenal mass. Table 1 represents the information and demographics of the patients.

**Table 1:** Patients’ demographics and workup

Patient	Age/gender	Weight (kg), BMI (kg/m^2^)	Comorbidities	Mass location/ size (cm)	Endocrine profile	Operative time (min)	Blood loss (ml)
Patient 1	45/M	117 kg, 41	HTN, DM	Right/ 4 cm	ACTH N S-ARR N S AM cortisol N S-M ↑ S-NM ↑	75 min	50 ml
Patient 2	55/M	131 kg, 40	IDDM, HTN, IHD, CKD	Right/ 1.7 cm	U-ARR ↑↑↑ U-M N UNM N	105 min	50 ml
Patient 3	37/M	111 kg, 42	HTN, DLD, gout	Left/ 5 cm	S-AM cortisol N S-M N S-NM N	80 min	20 ml

ACTH, adrenocorticotropic hormone; S-ARR, serum aldosterone-to-renin ratio; S-M, serum metanephrines; S-NM, serum normetanephrines; U-ARR, urinary aldosterone-to-renin ratio; U-M, urinary metanephrines; U-MN, urinary normetanephrines; IDDM, insulin-dependent diabetes mellitus; IHD, ischemic heart disease; CKD, chronic kidney disease; DLD, dyslipidemia.

### Patient 1

**Figure 1:**
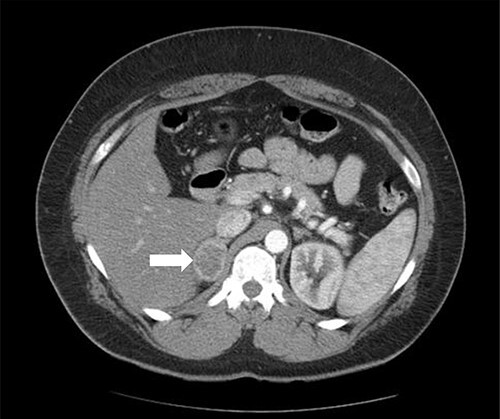
Axial CT of the abdomen showing the right adrenal mass in Patient 1 (white arrow).

Patient 1 is a 45-year-old male, who came to our private clinic complaining of obesity with a body mass index (BMI) of 41 kg/m^2^. His other comorbidities included hypertension (HTN) controlled on one anti-HTN medication and diabetes mellitus (DM) on oral hypoglycemic agents (OHA). He denied any symptoms of reflux or any family history of adrenal/thyroid/parathyroid diseases. He was discovered to have a 4 cm right adrenal incidentaloma on a contrast-enhanced computed tomography (CECT) of the abdomen as a workup for abdominal pain (Fig. 1). Preoperative preparation included obesity workup with complete blood count, coagulation profile and a chest X-ray. Endocrine workup included a screening for possible functional incidentaloma, with adrenocorticotropic hormone of 2.8 pmol/l, aldosterone/renin ratio (ARR) of 1.7 ng/dl per ng/(ml·h), serum metanephrine of 343 pg/ml (reference <90), serum normetanephrine of 401 pg/ml (reference <129) and a normal serum morning cortisol of 168 nmol/l (reference 101–535). A diagnosis of right adrenal pheochromocytoma was established. A cardiac consultation was done and determined that the patient is fit for surgery and formulated a preoperative medication with α- and β-blockers.

### Patient 2

**Figure 2:**
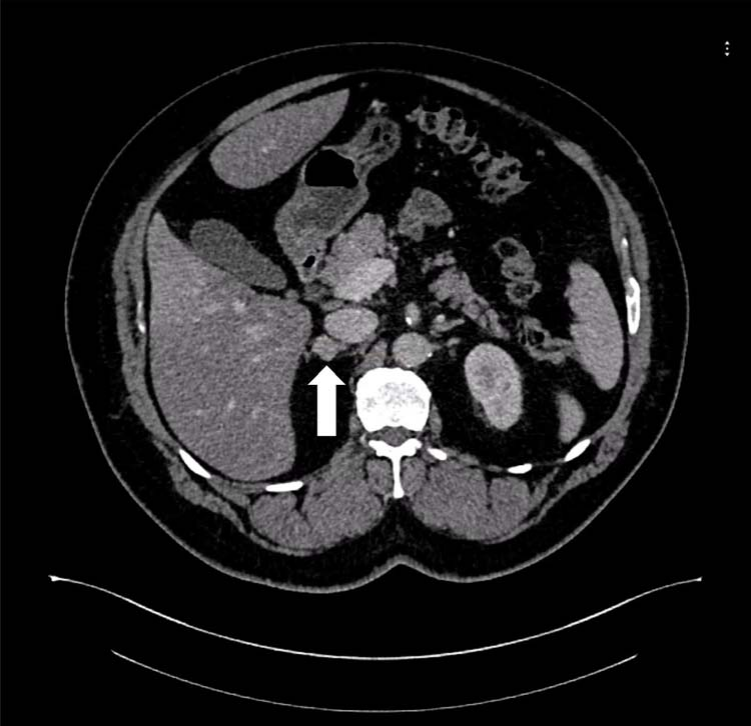
Axial CT of the abdomen showing the right adrenal mass in Patient 2 (white arrow).

A 55-year-old male, with a BMI of 40, was referred to our surgical clinic at a governmental/academic hospital after being investigated at endocrinology department. His medical history was significant for insulin-dependent DM, uncontrolled HTN on four medications, chronic kidney disease and ischemic heart disease with multiple coronary stents on antiplatelets. A biochemical workup showed an elevated serum aldosterone of 100 ng/dl, elevated serum renin of 109 uIU/ml and elevated urinary ARR of >7000 mg/g. Serum potassium and sodium levels were within normal limits. A CECT of the abdomen and pelvis confirmed an isodense right adrenal focal lesion measuring 1.7 × 1.6 cm in pre-contrast phase, with enhancement on contrast phase followed by rapid washout, concluding a diagnosis of Conn’s syndrome (Fig. 2). Peri-operative optimization of blood pressure/cardiac status and management of anticoagulation were done through cardiac consultation. Due to his cardiac status, the patient was labeled as high risk for peri-operative cardiac events. A specialized cardiac anesthetist was consulted to perform the anesthesia.

### Patient 3

**Figure 3:**
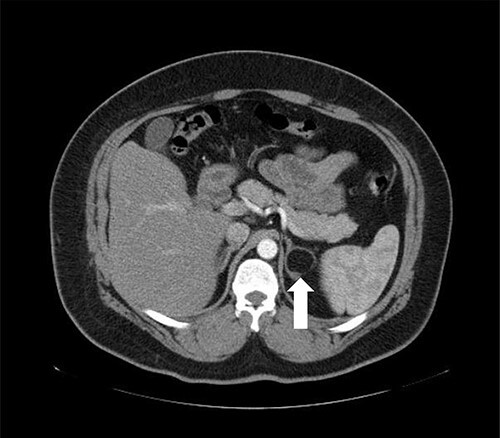
Axial CT of the abdomen showing the left adrenal mass in Patient 3 (white arrow).

A 37-year-old male, with a BMI of 42 kg/m^2^, dyslipidemia HTN and gout, presented to our private clinic with progressive weight gain. He also reported an incidental 1.6 cm left adrenal mass on a CECT of the abdomen, which was done in 2014. A repeated CECT showed a larger left adrenal mass of 5 cm, which was lobulated and suggestive of atypical lipid-poor adenoma (Fig. 3). A biochemical workup showed normal serum metanephrine of 50 ng/l (reference <90), normal serum normetanephrines of 90 ng/l (reference <129) and serum morning cortisol of 250 nmol/l. Considering the increasing size of the left adrenal gland and the patient's concerns about it, a LSG combined with LLA was offered to the patient for weight management and diagnosis confirmation of the left adrenal gland.

### The operation

Before shifting to the operative room, an anticoagulant is given 2 hours preoperatively. All surgeries were conducted by a Fellow of the Royal College of Physician and Surgeon of Canada trained surgeon, with over 30 bariatric surgery cases per week. At the operating room, patient positioning, application of pneumatic compression device and administration of antibiotic prophylaxis were done. After anesthesia induction, preparation of surgical filed was completed. Abdomen was entered using 5 mm vesiport superior and to the left of umbilicus.

**Figure 4:**
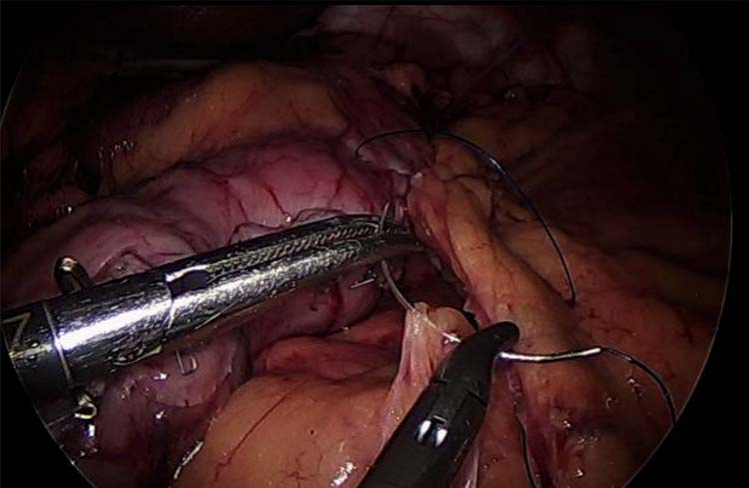
Omentopexy after gastrectomy.

For LLA, ports were inserted similar to our preferences for LSG, which are 5 mm at left upper quadrant, 12 mm at superior and to the right of umbilicus, 5 mm port at right upper quadrant and a Nathanson liver retractor at epigastric area. In Patient 3 with left adrenal mass, we proceeded with LSG first since the patient is not a high risk and that LSG will ease the LLA from exposure standpoint. The greater omentum was divided till the gastroesophageal junction, followed by applying 60 mm black Ethicon Tristapler at the antrum followed by purple staplers along a 36Fr bougie. It is our preference to apply clips long the sleeve but not a full deployment to control bleeding. Using the supragastric approach described before, the left adrenal gland was reached easily using the left diaphragmatic crus as a landmark and tracing the left phrenic vein to reach the left adrenal vein [7]. Using energy device, the left adrenal vein was controlled and attachments were released liberating the left adrenal gland and placed in the endobag. After assuring hemostasis, we completed the procedure by plicating the stomach at the upper half of the sleeve and omentopexy of the whole sleeve till the end of the divided omentum (Fig. 4).

**Figure 5:**
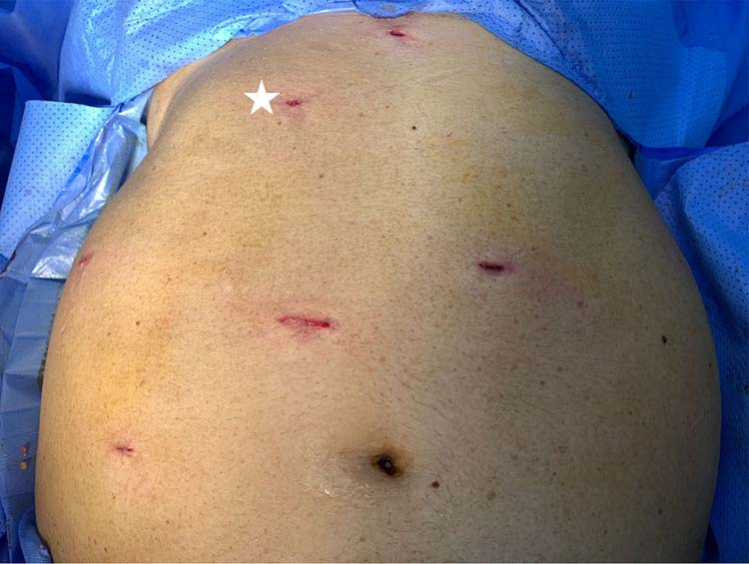
Port placement in Patient 2. The port marked with white star is an additional/optional port for better assistance in LRA.

**Figure 6:**
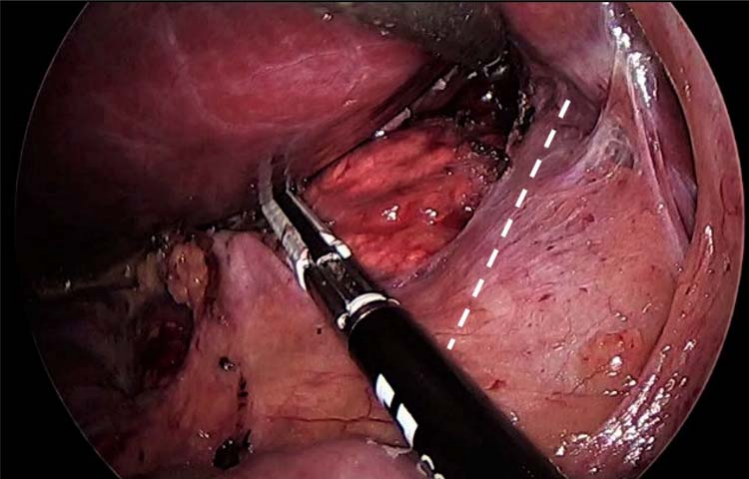
The right adrenal gland next to the IVC (dashed line).

**Figure 7:**
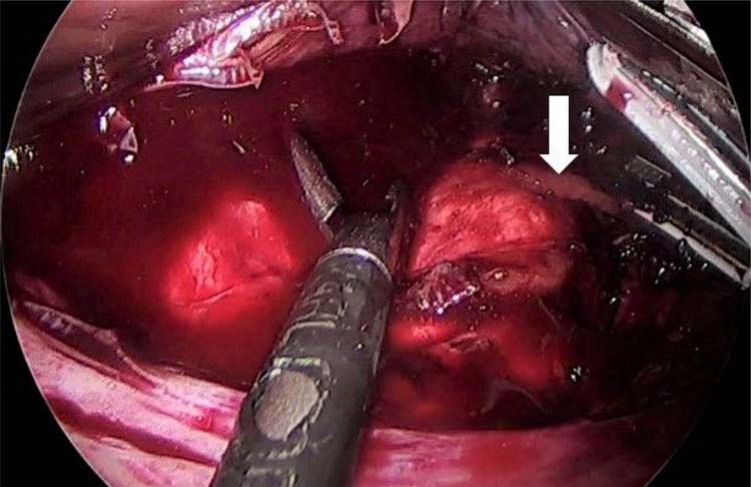
Right adrenal vein (white arrow).

**Figure 8:**
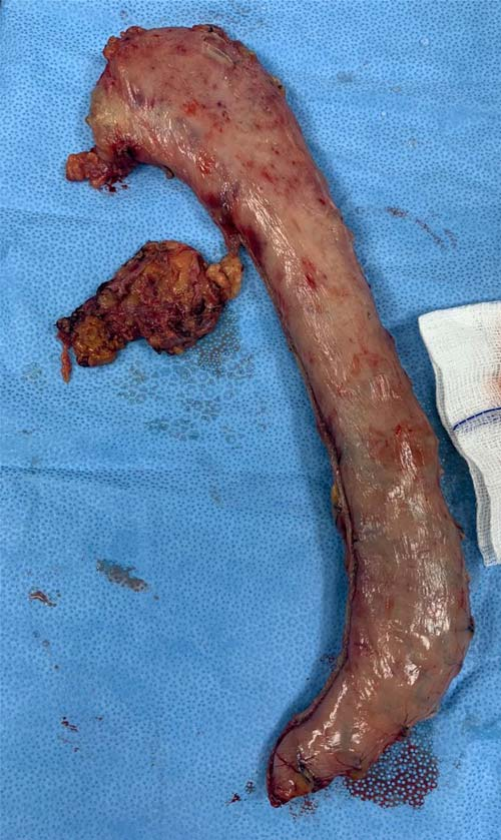
The extracted gastric sleeve and the right adrenal gland.

As for the LRA, ports are inserted in similar fashion as the LLA but with more to the right side. An additional port at the right midclavicular line is an optional (Fig. 5). In Patient 1, we started with the LLA to control the blood pressure caused by the adrenal gland and to prevent unnecessary bleeding from occurring. Conversely, we started with LSG in Patient 2 because the priority was controlling his comorbidities by weight reduction. The right hepatic lobe was retracted to expose the subhepatic area. The right hepatic ligaments were released, exposing the inferior vena cava. Dissection lateral to the IVC was continued exposing the right adrenal (Fig. 6). Complete dissection of the adrenal gland was completed and the pedicle was controlled using energy device (Fig. 7). There was constant communication with the anesthesia team during the surgery. After complete dissection of the adrenal gland, it was placed in the endobag and the LSG was conducted similar to the above-mentioned steps (Fig. 8).

## RESULTS

All patients tolerated the surgery with no immediate complications. We followed our established protocol for LSG where clear liquids are started 6 hours from surgery, utilizing incentive spirometry and encouraging ambulation. Pain medications, anticoagulants and antiemetics were administrated according to the protocol. All patients were monitored until the next day, visited by the managing team and the clinical dietician. Clear written instructions were given regarding the fluid intake, dietary advise, medications/vitamins, follow-up appointments and contact number for any emergency.

After 3 months of follow-up, Patient 1 had uneventful postoperative course and lost 25 kg. Patient 2 lost 20 kg, stopped three of his four anti-HTN medications, held insulin and had better glucose level readings. Unfortunately, he developed hyperkalemia because of contralateral adrenal suppression, which is managed by endocrinology through hormonal replacement (Clavien-Dindo grade 2). After 1 year post surgery, Patient 3 lost 32 kg and ceased his anti-dyslipidemia drugs. Due to his noncompliance with regard to diet and vitamins, Patient 3 developed fatigue and vitamin D deficiency, which were corrected by supplementation and dietary instructions (Clavien-Dindo grade 1). Both Patient 1 and Patient 3 stopped their anti-HTN medications and OHA. All patients expressed their satisfaction about the surgery and how it positively affected their quality of life. All patients labeled the surgery as successful and were pleased about the results (Table 2).

**Table 2:** Patients’ outcome

Patient	Length of follow-up	Weight loss	Comorbidities status	Pathology	Complications
Patient 1	3 months	25 kg	Stopped anti-HTN Stopped OHA	Pheochromocytoma	None
Patient 2	3 months	25 kg	One anti-HTN Stopped insulin Better glucose level	Conn’s disease	Hyperkalemia
Patient 3	1 year	32 kg	Stopped anti-HTN Stopped anti-DLD Stopped OHA Better HbA1c	Adrenal lipoma	Vit D deficiency

DLD, dyslipidemia.

## DISCUSSION

Adrenal incidentaloma is a condition diagnosed by imaging modalities for other reasons. The prevalence of AI reported in literature ranges from 1% to 4%. Increased utilization and advances in imaging modalities could be attributed to the varying reported rate of AI in the literature [8–10]. It is recommended to approach AI by appropriate investigations and decision for surgical management through established guidelines [11, 12]. With the advent of minimally invasive surgery, more adrenalectomies are performed laparoscopically. In a recent systemic review, LA showed superior results compared to open adrenalectomy with regard to intraoperative blood loss, blood transfusion, and hospital stay [13].

With a growing number of metabolic procedures, the odds of concomitant surgeries increased but also raised the question of its safety and feasibility. A recent review of the Metabolic and Bariatric Surgery Accreditation Quality Initiative Program (MBSAQIP) by Clapp and colleagues investigated the safety of laparoscopic cholecystectomy or hernia repair with metabolic surgery. In general, treating two surgical diseases in one setting seems more cost-effective, limits anesthetic adverse events and more convenient to the patient. Alternatively, a concomitant surgery had higher operative time (105–112 min for LSG + CS vs. 79 min for LSG alone), significant 30-day readmission rate (4.4-5% for LSG + CS vs 3.5 for LSG alone), and higher morbidity for pulmonary embolism (both <1%) and 30-day reoperation/intervention (both <2%).

The combination of MS and LA in one setting was reported before in the literature. Bardaro and Gagner performed a LLA during laparoscopic Roux-en-Y gastric bypass. The division of the stomach to form the pouch allowed a clear advantageous window accessing the adrenal gland. The procedure was completed with no immediate or late complications [14]. Another report by Soricelli *et al*. demonstrated the applicability and ease of LLA following LSG. The gland was broadly accessible and resection was completed smoothly [15]. De Gordejuela *et al*. performed a concomitant LSG with LLA due to Cushing’s disease. The method was similar to what Bardaro and Gagner done by the supragastric approach [16].

To our knowledge, this report is the first CS of LRA and LSG in the literature. This combination is slightly more challenging than a concomitant metabolic surgery with LLA for several reasons. Since the left adrenal gland is at proximity to the stomach, the port locations can be the same as LSG alone. Conversely, more planning for port locations is needed when performing RLA. The exposure of left adrenal gland is considerably superior since the field is opened after gastric division. Additionally, having a relatively constant course for the left adrenal vein makes the LLA relatively easier than LRA. After LSG, the LLA can be facilitated by following the left diaphragmatic curs followed by identifying the left phrenic vein where it can be traced to locate the left adrenal vein. When anatomical variations are encountered during LLA, it is typically in the form of joint drainage with the left phrenic vein or rarely gonadal vessels. On the other hand, anatomical variations during LRA are more diverse; the right adrenal vein can drain at any location along the inferior vena cava, with multiple branches, and alternatively can drain into the right hepatic, or the right renal vein [17]. In a review conducted by Scholten *et al*., although operative time was longer for the LLA, it was found that right adrenal gland had higher chances of anatomical variations compared to the left adrenal gland; hence more meticulous dissection is needed for LRA [18]. LA in patients with obesity has been associated with prolonged operative time and increased blood loss which could be due to hepatomegaly and excess retroperitoneal tissue [19, 20]. Weight reduction by metabolic surgery followed by LA as a second stage has been proposed in order to minimize operative difficulty caused by obesity [21].

Any AI should be investigated thoroughly for all possible causes, but most importantly to rule out hypercortisolism or Cushing’s syndrome. Undiscovered hypercortisolism can hinder the effect of metabolic surgery in patients with obesity. Fleseriu *et al*. reported two cases of undiagnosed Cushing’s syndrome who underwent unsuccessful metabolic surgery [22]. Suboptimal outcomes were also reported by Javorsky and colleagues had 10 out of 17 patients (one had adrenal adenoma) subjected for metabolic surgery without testing for hypercortisolism. From a weight reduction standpoint, trends for nadir BMI were higher in patients not treated for Cushing’s syndrome (median BMI of 38 kg/m^2^) compared to when treated (median BMI of 35 kg/m^2^). With regard to comorbidities resolution, only one patient had resolution of DM, and another patient stopped antihypertensive medications [23]. Despite that, the consensus does not support routine testing for hypercortisolism; thus, high index of suspicion is needed. We realize that this is a case series with small number of cases, but it shows that LSG and LA are feasible and doable when conducted by a well-trained laparoscopic surgeon and appropriate settings.

## CONCLUSION

With specific prerequisites, combining LSG with LA is safe and feasible. These requirements include a thorough workup, clear communication with the patient about risks and benefits, a well-trained specialized laparoscopic surgeon and a reliable anesthesia team. This concomitant surgery can be cost-effective and convenient for the patient when needed.

## ETHICAL APPROVAL

All procedures performed in studies involving human participants were in accordance with the ethical standards of the institutional and/or national research committee and with the 1964 Helsinki declaration and its later amendments or comparable ethical standards

Research registration: researchregistry7517

Guarantor: Dr Awadh Alqahtani

## INFORMED CONSENT

Written consent was obtained from all patients.

## AUTHORS' CONTRIBUTION

M.A.: Writing of paper, review of literature, collecting images. H.H.: Writing of paper, review of literature, collecting images. S.B.: Collecting images. A.A.: Conceptualization, review and editing

## CONFLICT OF INTEREST STATEMENT

None declared.

## FUNDING

None.
